# Subnational variations in electricity access and infant mortality: Evidence from Ghana

**DOI:** 10.1016/j.hpopen.2021.100057

**Published:** 2021-12-02

**Authors:** Mikidadu Mohammed, Mathias Akuoko

**Affiliations:** a Department of Economics & Business Administration, Austin College, 900 North Grand Avenue, Sherman, TX 75090, USA; b Public Health Department, Austin College, 900 North Grand Avenue, Sherman, TX 75090, USA

**Keywords:** Infant mortality, Electricity access, Subnational, Sustainable infant health, Education, Wealth distribution

## Abstract

**Objectives:**

The study investigated the relationship between electricity access and infant mortality at the subnational level in Ghana, controlling for correlates such as birth interval, children living with both parents, women’s education, and income distribution.

**Methods:**

The study employed a pooled cross-section regression approach using data from the Ghana Demographic and Health Survey (GDHS) for 10 administrative regions from 1993 to 2014. The GDHS is a detailed data set that provides comprehensive information on households and their socio-economic and demographic characteristics in Ghana.

**Results:**

The results show that in regions with low incidence of infant mortality, a 10% improvement in electricity access reduces infant death by 11.8 per 1,000 live births, whereas in high mortality regions, improvement in electricity access has no impact on infant death. Birth interval reduces the risk of infant death in low mortality regions but not in high mortality regions. Children living with both parents have a high probability of survivorship in high mortality regions. Women’s median years of education lowers the likelihood of infant death in high mortality regions but increases the likelihood in low mortality regions. Wealth distribution is inconsequential for infant death in low mortality regions, but in high mortality regions, both the wealthiest and the poorest experienced significant decline in infant death.

**Conclusions:**

The findings underscore the fruitfulness of investigating the effects of electricity access and other correlates on infant mortality at the subnational level. The study recommends that the provision of reliable access to electricity is needed to improve infant mortality rates. However, policies that seek to improve access to reliable electricity should be implemented together with health infrastructure development policies, especially in the regions with high infant mortality rates, for electricity access to have the desired effect.

## Highlights

• Infant mortality is endemic in Ghana and varies significantly across regions.
• Electricity access reduces the likelihood of infant death in low mortality regions.
• Socio-economic correlates have differential effects across regions.
• A national program that integrates infant health and electricity access is necessary.

## 1 Introduction

Every year, 4.6 million children die before their first birthday in the world [1]. A woman in sub-Saharan Africa has a 1 in 45 chance of dying while giving birth—in the developed world, the chance is 1 in 5,400 [2]. In Ghana alone, more than 41,000 children die before their first birthday [3]. A number of studies have examined the underlying causes of infant mortality in Ghana. These include multiple-gestation, birth weight, grand multiparous mothers, breastfeeding, antenatal and postnatal services, and socioeconomic deprivation [4], household wealth, spacing, and parental education [5], birth interval and unprotected water sources [6]. Other predictors are female education [7], [8], [9], mothers’ age, and marital status [10], early infant feeding practices [11], rural–urban migration patterns [12], family structure [13], infectious diseases [14], previous child death, vitamin A supplements, exclusive breastfeeding, number of children, and immunization [15], [16]. While these studies provided important insights about the drivers of infant mortality in Ghana, their focus on the drivers of infant death at the national level mask important variations at the subnational level. Furthermore, as critical as electricity access is to health outcomes, studies that examined the relationship between electricity access and infant mortality in Ghana are rare. Infant mortality is endemic in the Ghanaian society and varies significantly across regions. Given the country’s epileptic electricity generation and distribution, a national program that integrates infant health and electricity supply services would be urgent if a strong association between infant health and electricity access were established.

## 2 Background

The quantity and quality of energy consumed is to a certain extent predictive of life expectancy and other health outcomes [17], [18]. Theoretically, energy consumption affects health outcomes through three main channels: fossil fuel-related air pollution, extent of urbanization, and the degree of energy access. There is a fairly sizeable empirical literature showing that these channels produce benefits (risk factors) that improves (deteriorates) the health conditions in a given geographical location [19], [20], [21], [22], [23], [24], [25], [26], [27], [28], [29], [30], [31], [32], [33], [34], [35], [36], [37], [38], [39], [40].

While most of the studies investigating the relationship between energy consumption and health outcomes have been conducted using total energy consumption, there is some research that investigates the relationship between electricity access, a critical energy form, and health outcomes such as infant mortality, life expectancy, and air pollution. Examining the relationships among electricity usage, coal consumption, and health outcomes in 41 countries, one study found that increased electricity consumption is associated with reduced infant mortality for countries that started with relatively high infant mortality rates, whereas life expectancy is not significantly associated with electricity consumption [41]. In a 2014 study that examined the effect of energy consumption-related air pollution on pre-mature deaths around the world, the authors found that 4.3 million people die a year prematurely due to household air pollution caused by the use of less clean energy forms [24]. The study also noted that nearly half of deaths due to pneumonia among children under-five years of age are caused by particulate matter (soot) inhaled from household air pollution. Another study estimating the quality of life as a function of electricity consumption [42] concluded that per capita electricity consumption is positively associated with life expectancy and inversely related to infant mortality.

In developing countries, lack of electricity access has been associated with worsening infant mortality rates because it stimulates the use of inefficient energy forms such as kerosene, biomass (wood, animal dung and crop waste), and charcoal. Given that women are usually responsible for cooking while caring for infants, women and infants are most exposed to indoor air pollution that increases morbidity and mortality [43]. Moreover, lack of electricity disincentivizes doctors and nurses from taking jobs in areas that desperately need maternal and neonatal medical attention, which also contributes to poor health outcomes [25]. Lack of electricity is also associated with lack of hygienic conditions and inability to conserve drugs for extended usage. Improvements in electricity access enhance the provision of clean drinking water and better refrigeration that enable drug and food conservation thereby reducing the incidence of infant mortality [44], [45]. Furthermore, critical lack of functional lighting in health facilities offering child delivery and night-time care increases the incidence of infant mortality [46].

Despite the importance of electricity access to health outcomes, studies that examined the relationship between electricity access and infant mortality at the subnational level in Ghana are non-existing. The only exception is a 2019 study in which the author examined the effects of energy use on infant mortality rates at the subcontinent level consisting of 23 African countries including Ghana [40]. Even so, the study focused on each country as a whole and not their subnational administrative regions. Furthermore, the study used total energy consumption and not electricity access. One reason for the scarcity of studies that examined the electricity access-infant mortality relationship at the subnational level is the lack of consistent disaggregated data on predictors of infant mortality. The other reason is the assumption that infant mortality rate, which have been falling at the national level is also falling at the subnational level.

However, besides electricity access, subnational analysis about the role of other determinants of infant mortality in Ghana do exist and their findings suggest that analyzing the determinants of infant mortality at the national level masks important variations at the disaggregated level. For example, a 2003 study used linear regression models to examine the impact of mothers’ education on childhood mortality [7]. The study found that there is an inverse relationship between mothers’ education and childhood death. Also, the study finds significant differences in mortality by place of residence. Childhood mortality at every level is lower in the urban than the rural areas.

Examining the socio-economic and demographic determinants of under-five mortality in rural northern Ghana, another study found similar results for mothers’ education [10]. Specifically, mothers with primary or junior high school education were 45% less likely to experience under-five death than mothers with no formal education. In addition, older women (ages of 35 and 49 years) were more likely to experience under-five death than younger women whereas married women had a low probability of experiencing an under-five death than those who were single, divorced or widowed [10].

Using multi-level analysis, a 2014 study investigated the individual and community determinants of under-five mortality in Ghana and found that infants of multiple-gestation, neonates with inadequate birth spacing, low birth weight, infants of grand multiparous mother, and non-breastfed infants had a lower chance of surviving the neonatal period [4]. In addition, adequate utilization of antenatal, delivery and postnatal healthcare reduced the probability of neonatal mortality whereas dwelling in a neighborhood with high socioeconomic deprivation is associated with increased infant mortality.

According to another study that implemented a proportional hazards model to estimate variations in infant mortality driven by rural–urban migration patterns, infant mortality is lower among rural–urban migrants compared to rural non-migrants [12]. In a randomized controlled neovita trial in the Brong Ahafo region of Ghana, a 2008 study rejected the inclusion of newborn vitamin A supplementation as a child survival strategy in Ghana [11]. Family structure is also recognized as an intervening factor in accounting for infant survival in Ghana [13].

Beyond studies that focus on Ghana, other correlates of infant mortality identified in the literature for national and subnational levels include presence of parents and other family members, season of a child’s birth, sex of the child, age of mother at birth, multiple versus single births, birth location, birth rank, the number of siblings, mortality of older siblings, and mothers’ occupation in India and China [47], [48], [49], inequality in 145 countries [50], primary care physician supply in the US states [51], wages and housing density in New Zealand [52], public spending on health, GNI/capita, poverty rate, income equality, and young female illiteracy rate in 152 countries [53]. Additional correlates include neighborhood sanitation in India [54], income distribution is six countries in West Africa [55], access to improved sources of drinking water, improved toilet facilities, antenatal care, and skilled delivery in Nigeria [56], access to primary care and conditional cash transfers in Brazil [57].

The subnational studies [47], [48], [51], [52], [51], [56], [57] revealed significant cross-regional disparities in the determinants of infant mortality that the national studies [49], [50], [53], [55] could not have accounted for. For instance, the subnational study in rural India [48] found that the infant mortality rate for males was 113 and that for females was 90. The difference between the two rates is significant. However, for the country as a whole, a survey conducted by the Registrar General revealed a higher infant mortality rate for females (131) than for males (120); but the rates for the two sexes were the same (71) for urban areas. The implication is that without the subnational analysis, policy makers would not have known what the rural infant mortality rate for females and males were and how they differ from the urban areas. Another subnational study on US states concluded that health inequalities among population groups across states continue to increase despite national commitments to reduce them [51]. Yet another subnational study examined the combined effects of access to primary care and conditional cash transfers infant mortality for 4,583 Brazilian municipalities [57]. The author concluded that the effect of access to primary care depends on the expansion of the conditional cash transfers and that impoverished, underserved populations react differently from wealthy, well-catered for populations. For the impoverished, underserved populations, the author recommended policies that combine supply- and demand-side interventions to mitigate the adverse effects on the disadvantage populations. Collectively, these subnational analyses show how national level analysis of drivers of infant mortality in other countries have masked important variations at the subnational level and the implications thereof. They also signify that it is important to examine the trends and determinants of infant mortality in different parts of a country so that special attention can be given to areas where infant mortality continues to be high.

The above clearly shows that although several studies have found significant relationship between electricity access and infant mortality, evidence regarding the impact of electricity access on infant mortality rates at the subnational level in Ghana is non-existing. Therefore, this study seeks to investigate: (1) whether rising (falling) electricity access reduces (increases) the rate of infant mortality in Ghana and (2) whether improved electricity access does affect infant mortality rates at the subnational level without *a priori* hypotheses as the nature of the effects. In addition, the literature identified several important correlates of infant mortality. This study also includes the control correlates insofar as data are available.

## 3 Study area

**Fig. A1:**
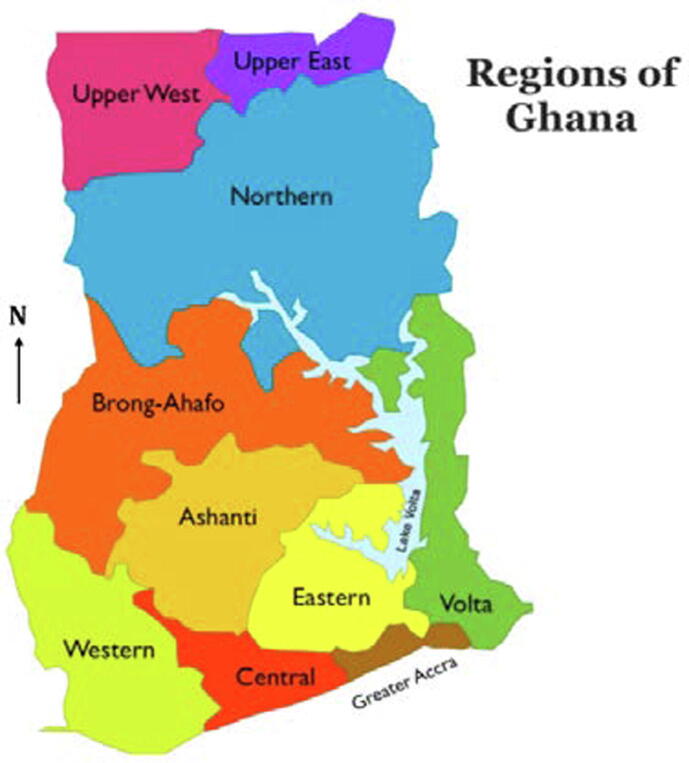
Map of Ghana with various administrative regions. Source: scooper maps.

Ghana is located in West Africa. It borders Togo in the east, Ivory Coast in the west, and Burkina Faso in the north. In the south is the gulf of guinea which opens into the Atlantic Ocean. The country has a total land area of 239,460 km^2^ and a water area of 8520 km^2^ [58]. The country’s population size is about 27.8 million as at 2015 with an annual growth rate of about 2.6% [59]. Ghana’s climate is tropical and strongly influenced by the West African monsoon winds. The northern part of the country is dry and arid, and the southern part is comparatively warm and humid. Ghana had 10 administrative regions prior to 2019. Following a referendum in 2018, the administrative regions were redrawn to become 16 regions in 2019. To be consistent with the Ghana Demographic and Health Survey (GDHS) data that ends in 2014, this study uses the old 10 regional map of Ghana. These include Ashanti, Brong Ahafo, Central, Eastern, Greater Accra, Northern, Upper East, Upper West, Volta, and Western (Fig. A1 in the Appendix).

Electricity transmission network in Ghana connects electricity generation sites at Akosombo, Kpong, Aboadze and Tema to the various load centers in the country. The network is made up of more than 40 primary substations (transformation and switching substations), linked by over 4315.5 km of high voltage transmission lines [60], [62]. According to the Ghana Wholesale Power Reliability Assessment Report, approximately 3,888.1 km and 132 km of the transmission system are energized at 161 kV and 69 kV, respectively. In addition, 73.4 km of the transmission is also energized at 225 kV. The current total installed transformer capacity of the network is 2,915 MVA [64], [65]. The high transmission voltages are reduced to 34.5 kV, 11.5 kV and 6.6 kV at the primary substations for supply to bulk customers and/or onward distribution to end users throughout the country [60].

**Fig. 1:**
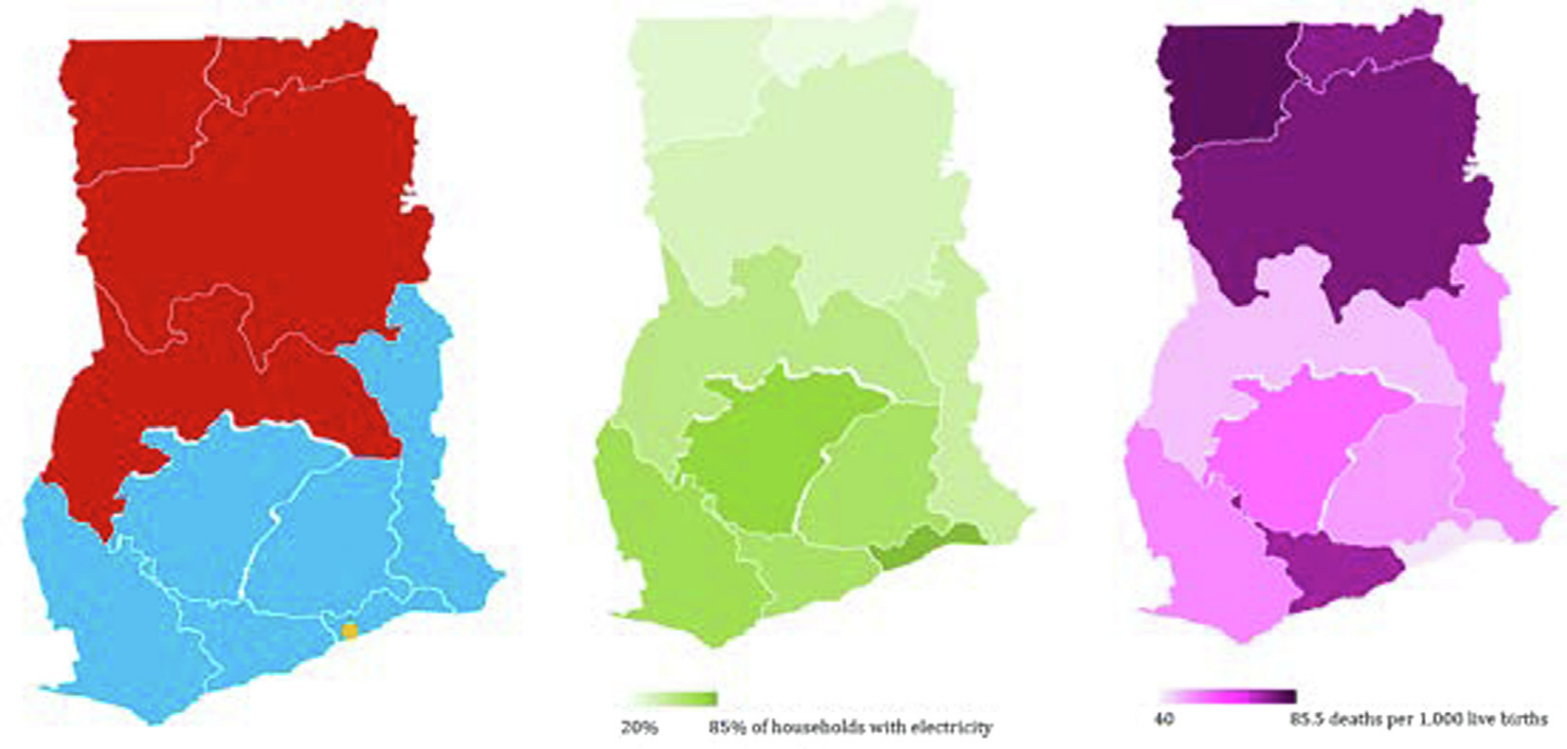
(a) Electricity Distribution Zones: ECG (blue), NEDCo (red), and EPDC (yellow). Source: Ghana Wholesale Power Reliability Assessment Final Report 2010. (b) Regional distribution of electricity access (Averages for 1993–2014). “Regional distribution of electricity access (Averages for 1993–2014).”: The darker the green, the higher the concerntration of electricity access. Source: Ghana Demographic and Health Survey and Authors’ calculation. (c) Regional distribution of infant mortality per 1,000 live births (Averages for 1993–2014). Low infant mortality regions (40–57.5 infant deaths per 1,000 live births). High infant mortality regions (60–84.5 infant deaths per 1,000 live births). Source: Ghana Demographic and Health Survey and Authors’ calculation. (For interpretation of the references to colour in this figure legend, the reader is referred to the web version of this article.)

Electricity distribution in the country is undertaken by two public distribution companies and one private distribution company (Fig. 1 panel (a)). The two public companies are Electricity Company of Ghana Ltd (ECG) and Northern Electricity Distribution Company Ltd (NEDCo) [63]. The private distribution company is Enclave Power Distribution Company Ltd (EPDC). ECG distributes electricity in the southern sector of the country to a total customer population of 2.1million as of the end of 2010 [66]. NEDCo distributes electricity in the northern sector of the country to a total population of 342,207 [67]. EPDC has been providing electricity supply primarily to industrial and commercial customers within the Free Zones Enclave in Tema, in the Greater Accra Region [61].

In terms of nationwide electricity policies, the Ministry of Energy formulates the main policies that govern the electricity sector. The Ministry is also responsible for monitoring and evaluating policies, programs, and projects in the electricity sector. There is also another important entity called the Energy Commission, a quasi-independent body established by the Energy Commission Act of 1997 (Act 541). The Commission is the government’s energy policy advisor and makes national energy policy recommendations to the Minister of Energy. Specifically, the Commission advises the Minister of Energy on national energy policies for the efficient, economical, and safe supply of electricity and natural gas, especially as they relate to the growth and development of the country. In addition, the Commission formulates national policies for the promotion and utilization of local renewable energy such as solar, wind and biomass [60], [64], [65].

Since the mid-1990 s, there has been a shift in the electricity sector policy direction. The electricity sector was reformed to attract private sector investment and state of the art electricity generation technology. As part of the reform, the Volta River Development Act (Act 61), which established the Volta River Authority, was revised in 2005 into the Volta River Development Amendment Act, Act 692 [66]. The new Act 692 transferred the electricity transmission function of Volta River Authority (VRA) to a new company, Ghana Grid Company Ltd (GRIDCo), incorporated in 2006 as a private liability company under the Companies code 1963, Act 179. GRIDCo is expected to provide fair and non-discriminatory transmission services to all electricity market participants [67].

The reform policy in the electricity sector gained momentum in the mid-2000s with the aim of establishing a wholesale electricity market to promote competitive electricity pricing. In 2008, Parliament enacted Wholesale Electricity Market — Technical Rules (Legislative Instrument No. 1934) and Wholesale Electricity Market Operational Regulations — (Legislative Instrument No. 1937), which effectively establish the wholesale electricity supply market.

The retail consumption sector is divided into three primary and two secondary markets [68]. The primary markets include the industrial, residential, and non-residential. Each market faces a different tariff regime. The industrial market are consumers who use electricity for industrial purposes. Residential consumers refer to homes in both rural and urban sectors of the country, although skewed heavily towards the urban areas. Non-residential consumers are mostly commercial facilities. The two secondary markets are street lighting and export. The electricity consumed by streetlights across the country is captured under the street lighting class. Exports of electricity are to neighboring countries of Togo, Ivory Coast, and Burkina Faso. Fig. S1 in the supplementary materials shows the electricity consumption trend in Ghana since 2006. Clearly, the industrial sector, residential sector, and non-residential sector emerge as the three top consumers of the electricity in the country. Export and street lighting on the other hand account for the smaller proportion of electricity consumption. What is surprising is that the amount of electricity lost in transmission and distribution has been on the increase since 2006 in a country where 84% of urban households have access to electricity whereas only 40% of rural households have access [68], [69].

Electricity access is defined as percentage of households with electricity. Fig. 1 panel (b) shows the distribution of electricity access across the regions in the country. The data is obtained from the Ghana Demographic and Health Survey (GDHS) database. Estimates are given for 5-year intervals: 1993, 1998, 2003, 2008, and 2014. As observed from the figure, electricity access varies significantly in Ghana. Regions with low access are concentrated in the northern part of the country (Upper East, Upper West, and Northern) whereas regions with the most access are concentrated in the south (Western, Ashanti, and Greater Accra). The regions in the north are predominantly rural whereas the regions in the south, especially Greater Accra and Ashanti, are predominantly urban.

The healthcare system of Ghana is mostly controlled by the government. Through the Ministry of Health and the Ghana Health Services, the national government controls most of the healthcare system, including the financing and delivery functions. There is also private health sector that provides services to those who have the ability to pay. Funded through taxes, the government, through budgets, decides the amount of healthcare to be produced and delivered, and the number of healthcare infrastructure to be developed every year. The system is organized under five levels, from the Tertiary hospitals, through regional hospitals, district hospitals, clinic and health centers, to the health posts [70]. The further away you move from the urban centers, the more you move away from the tertiary hospitals to the health centers. The health posts are mostly located in rural and under resourced areas, and are in many cases staffed by community nurses and midwives.

The system continuous to face both financial and human resources challenges due to inadequate healthcare providers and financing [71]. Notwithstanding these, Ghana in 2003, established a National Health Insurance Scheme (NHIS) to provide access to an affordable healthcare to all Ghanaians [72]. However, the system still relies heavily on a cash-and-carry system where patients have to pay out of pocket for many of the services, medications, and some basic needed medical equipments. A lot of the private sector providers also do not accept the NHIS due to delay and low reimbursements rates. These have affected health care access to many Ghanaians, and patient satisfaction as well [71].

For those that do not have the resources, especially those in rural and underserved communities, they find it very difficult to access basic healthcare needs such as prenatal care, care during the birthing process, and care for infants [71]. All these factors contribute to rising infant mortality rates in the country [73].

Infant mortality rate is defined as the probability of dying before the first birthday in the five years preceding the survey, per 1,000 live births. Fig. 1 panel (c) shows the distribution of infant mortality per 1,000 live births across the regions in the country. The data is also obtained from the GDHS database. Infant mortality is widespread in the Ghanaian society and varies significantly across regions: higher in the three regions in the north, as well as Central and Western, but relatively lower in Greater Accra, and most of mid-southern regions.

Comparing Fig. 1 panels (b) and (c), it appears that the regions that show the least access to electricity are also the ones that show the highest incidence of infant mortality, and vice versa. The objective of the study is to examine whether these cross-regional variations in infant mortality rates can be explained by variations in electricity access.

## 4 Data and methods

### 4.1 Data

The study relied on the Ghana Demographic and Health Survey (GDHS), a national survey covering all ten regions of the country. The survey is designed to collect, analyze, and disseminate information on housing and household characteristics, education, maternal health and child health, nutrition, family planning, gender, energy access, socio-economic status and several other topics of investigation.

The GDHS program commenced in 1988 and is modeled in a standard demographic and health survey format. The unit of analysis is household, and characteristics of the household include children under five years, women age 15–49, and men age 15–59. Four main questionnaires are used to collect the data: Household Questionnaire, Woman’s Questionnaire, Man’s Questionnaire, and Biomarker Questionnaire.

The Household Questionnaire is designed to collect some basic information on the characteristics of each person listed, including his/her age, sex, education, and relationship to the head of the household. The primary goal of the Household Questionnaire is to provide the mechanism for identifying women eligible for individual interview and children under five who are to be weighed, measured, and tested for anemia.

The Woman’s Questionnaire is the central part of the DHS questionnaires and covers all of the key topics of the survey. These are background characteristics (age, education, religion, etc.), reproduction, contraception, pregnancy and postnatal care, child immunization, child health and nutrition, marriage and sexual activity, fertility preferences, husband’s background and woman’s work, HIV/AIDS, and other health issues. In addition to questions about the woman, the questionnaire contains a birth history that is used to list all children (alive or dead) that the respondent has given birth to, with the child’s sex, date of birth, age, and survival status. The birth history is then the basis for selecting children under certain ages for the maternal health, immunization, child health, and nutrition sections of the questionnaire.

The Man’s Questionnaire is similar but shorter than the Woman’s Questionnaire and is used to collect data on background characteristics, reproduction and fertility preferences, contraception, employment and gender roles, HIV/AIDS, and other health issues.

The Biomarker Questionnaire collects biomarker data for eligible household members. These include anthropometric measurements (height and weight), tests for the hemoglobin level in blood for anemia, malaria testing and other lab-based biomarkers. In earlier versions of the survey, the biomarker questionnaire was part of the Household Questionnaire.

The GHDS data is typically released in 5-year intervals: 1988, 1993, 1998, 2003, 2008, and 2014. Each release contains data on several variables. Users of the survey data can then use the STATCOMPLIER from the global DHS database to extract the variables. This study extracted all the data set for Ghana but because the 1988 release did not contain consistent data for the variables of interest, the study focused on the period 1993, 1998, 2003, 2008, and 2014 for the analysis.

The variables the study included are infant mortality rate defined as the probability of dying before the first birthday in the five years preceding the survey, per 1,000 live births; electricity access defined as percentage of households with electricity; median birth interval defined as median duration of the preceding birth interval (in months) for non-first births; children living with both parents measured as percentage of de jure children living with both parents; median number of years of education completed by women; and two wealth quintiles, (lowest and highest), defined as percentage of the de jure population in each category

### 4.2 Methods

The objective of the study was to examine whether subnational variations in infant mortality rates could be explained by variations in electricity access. Thus, our exposure variable was electricity access whereas our outcome variable was infant mortality rate. In addition, the literature identified several important correlates of infant mortality. The study included these correlates in the model estimations insofar as data were available. Specifically, we used two criteria. One, what control correlates are suggested by the literature? Two, is data available on the suggested control correlates? In this study, the correlates median birth interval, children living with both parents, median number of years of education completed by women, and the two wealth quintiles, (lowest and highest) met the two criteria and therefore were included in the model.

To better appreciate the relationship between electricity access and infant mortality, the study first compared and contrasted the two variables at the national and subnational level. The study also compared and contrasted each of the correlates with the variable electricity access and their variations or otherwise.

Empirically, the study utilized a pooled cross-section ordinary least squares (OLS) regression model that included infant mortality rate as the dependent variable and electricity access as the main explanatory variable together with other correlates of infant mortality as controls. The pooled cross-section regression approach is suitable for this analysis because the GDHS data has both cross-sectional and time series dimension since it is obtained by sampling randomly from a large population at different points in time. Our empirical model takes the following form:

(1) $$\mathrm{IM}R_{\mathrm{it}}=\beta PHHE_{\mathrm{it}}+\psi Z_{it}+\alpha_{i}\;+\delta_{t}\;+e_{\mathrm{it}}$$

where *i* indexes regions and *t* indexes time period; *IMR* denotes infant mortality per 1,000 live births; *PHHE* is percentage of households with electricity access; *Z* is a vector of control variables that include median birth interval (*MBI*), children living with both parents (*PCLP*), median number of years of education completed by women (*MYEW*), and two wealth quintiles, lowest (*PPLQ*) and highest (*PPHQ*); *α_i_* denotes region fixed effects, *δ_t_* denotes time fixed effects, and *e_it_* is the error term. All variables were transformed into first difference and were stationary at the 5% significance level according to the Augmented Dickey-Fuller and Phillips-Perron tests.

The estimation process was implemented as follows. First, the study estimated a time-series version of model (1) using the national data to examine the effect of electricity access on infant mortality at the national level. The sample was then split into two (low infant mortality regions and high infant mortality regions) and model (1), which was the pooled cross-section model, was estimated for each sub-sample to examine whether variations in electricity access triggered different infant mortality outcomes in high and low regions. The national infant mortality average of 61.38 infant deaths per 1,000 live births from 1993 to 2014 was used to determine the sample split. Regions with below average infant deaths were classified as low infant mortality whereas regions with above average infant deaths are classified as high infant mortality. Because the sample average of 61.3 deaths per 1,000 live births in the main estimated model was specifically for Ghana, the study checked the robustness of the results by using the sub-Saharan Africa 2018 infant mortality rate of 58.3 deaths per 1,000 live births. The sub-Saharan Africa benchmark rate was obtained from the World Bank World Development Indicators, which is more generalizable than the Ghana benchmark. The study however could not use the Millennium Development Goals (MDGs) target of 29 deaths per 1,000 live births or the Sustainable Development Goals (SDGs) target of 12 deaths per 1,000 live births [74] because the minimum infant mortality rate of 33 deaths per 1,000 live births in Ghana between 1993 and 2014 was above the two targets for the years under study.

## 5 Results

**Fig. 2:**
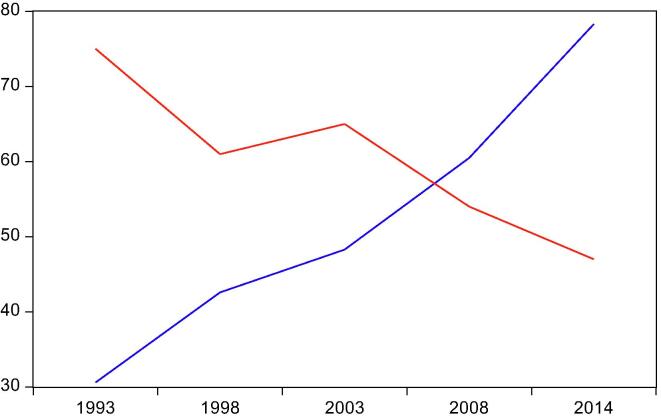
Infant mortality rates and electricity access in Ghana from 1993 to 2014. Infant mortality rate (red line) is defined as the probability of dying before the first birthday in the five years preceding the survey, per 1,000 live births. Electricity access (blue line) is defined as percentage of households with electricity. Data are obtained from the Ghana Demographic and Health Survey (GDHS). Estimates are given for 5-year intervals: 1993, 1998, 2003, 2008, and 2014. (For interpretation of the references to colour in this figure legend, the reader is referred to the web version of this article.)

**Fig. A2:**
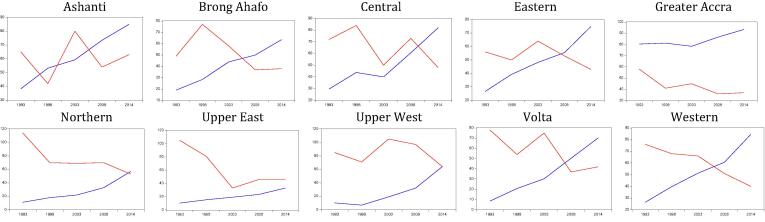
Regional variation in infant mortality rates and electricity access in Ghana (1993–2014). Infant mortality rate (red line) is defined as the probability of dying before the first birthday in the five years preceding the survey, per 1,000 live births. Electricity access (blue line) is defined as percentage of households with electricity. Same data sources as in Fig. 2. (For interpretation of the references to colour in this figure legend, the reader is referred to the web version of this article.)

The relationship between infant mortality and electricity access in Ghana is depicted in Fig. 2. Clearly, there is an inverse relationship between the two variables: infant mortality rate has been falling with rising electricity access. However, this masks the significant variations across regions in the country as depicted in Fig. A2 in the Appendix. For instance, in the Ashanti region, infant mortality rate has fluctuated despite persistent rise in access to electricity. In contrast, the Greater Accra region has experienced falling infant mortality rate with rising electricity access. This positive divergence accelerated in 2003. Other regions, such as Northern and Upper West, show slow convergence between infant mortality and rising electricity access, whereas Western, Volta, Eastern, and Brong Ahafo regions witnessed complete reversal in the relationship between infant mortality and electricity access. Moreover, among all regions, Northern, Upper East, and Upper West have the highest incidence of infant death. The three regions also show the least access to electricity. Could these cross-regional variations in infant mortality rates be explained by variations in electricity access? Do other factors play a role in the electricity access-infant mortality relationship?

Figs. S2 to S6 in the supplementary materials plot the relationship between each of the control correlates with the variable electricity access. As shown in Fig. S2, birth interval does not seem to vary with rising electricity access across the regions. Regarding the percentage of children living with both parents, most regions appear to experience falling rate of children living with both parents with rising access to electricity especially in the Eastern, Upper West, and Upper East, and Western regions (Fig. S3). However other regions such as Ashanti and Greater Accra seem to experience a rise in the percentage of children living with both parents as access to electricity increases. These same two regions also have their percentage of households with electricity consistently above the percentage of children living with both parents throughout the sample period.

Median years of women’s education does not seem to vary with rising electricity access across the regions (Fig. S4). For some regions, the median years of women’s education is either zero (Northern, Upper East, and Upper West) or less than 6 years (Volta and Western).

Wealth distribution at the lowest quintile appear to move in opposite direction with electricity access for some regions, particularly those in the south (Ashanti, Brong Ahafo, Central, Eastern, Greater Accra, Volta, and Western) whereas in the north (Northern and Upper East), the population in the lowest quintile moves in the same direction with electricity access (Fig. S5). In the north, only Upper West has seen some decline in population in the lowest quintile with rising electricity access.

Unlike the wealth distribution at the lowest quintile, the pattern of wealth distribution at the highest quintile does not exhibit variability with rising access to electricity (Fig. S6). The few exceptions are Upper East and Western where the percentage of the population in the highest quintile declines with rising electricity access throughout the sample period. Greater Accra also exhibits declining population in the highest quintile although much later, 2008 onwards.

Overall, the picture that emerged from comparing and contrasting each of the correlates with the variable electricity access is that not much variation exists between them across the regions, except for percentage of children living with both parents and wealth distribution at the lowest quintile. This is consistent with the multicollinearity test as shown in Table A1 in the appendix.

Summary statistics of all variables (outcome, exposure, and correlates) are provided in Table 1. We now turn our attention to the regression estimation results.

**Table 1:** Summary statistics of variables.

	**IMR**	**PHHE**	**MBI**	**PCLP**	**MYEW**	**PPLQ**	**PPHQ**
Mean	61.38	45.05	38.92	54.31	2.69	25.73	16.02
Median	58	41.9	38.2	50.55	2.45	19.15	11.65
Maximum	114	93.4	47.7	77.8	8.1	78.5	64.3
Minimum	33	7	33.5	36.3	0	0.6	1.1
Std. Dev.	19.52	24.71	2.96	13.08	2.24	22.45	16.60
Skewness	0.7164	0.2583	0.6637	0.6751	0.3743	0.9587	1.9178
Kurtosis	3.0255	1.9035	3.3168	2.0561	2.2515	2.5715	5.8237

Note: IMR is infant mortality rate defined as the probability of dying before the first birthday in the five years preceding the survey, per 1,000 live births, PHHE is percentage of households with electricity, MBI is median duration of the preceding birth interval in months for non-first births, PCLP is percentage of de jure children living with both parents, MYEW is the median number of years of education completed by women, PPLQ is percentage of the de jure population in the lowest wealth quintile, and PPHQ is Percentage of the de jure population in the highest wealth quintile. The data are obtained from the Ghana Demographic and Health Survey (GDHS). Estimates are given for 5-year intervals: 1993, 1998, 2003, 2008, and 2014.

**Fig. A3:**
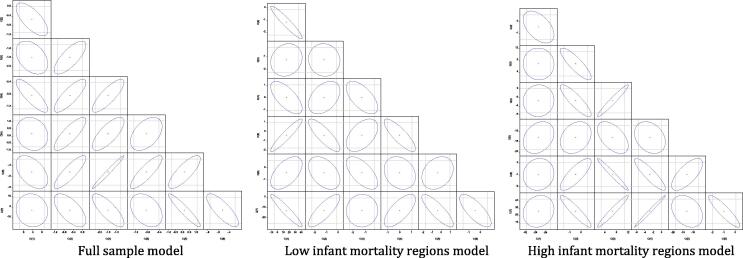
Multicollinearity Test (Confidence Ellipses). Note: For each model, circular and small eccentric elliptical confidence contour indicates variables are uncorrelated, and tighter/large eccentric elliptical contour indicates positive or negative correlation between variables. The presence of fewer tighter/large eccentric elliptical contours in each model indicates absence of multicollinearity.

Table 2 reports the regression estimation results. The Full sample model (1) reports the results when all regions were included in the estimation -. Because of the heterogeneity across the diverse set of regions, the Low infant mortality regions model (2) and the High infant mortality regions model (3) compare the results when the sample was restricted to low infant mortality rate regions and high infant mortality rate regions. The sample average of 61.3 deaths per 1,000 live births (1993–2014) was used to split the sample into two: low infant mortality regions (average deaths per 1,000 live births ≤ 61.3) included Ashanti, Brong-Ahafo, Eastern, Greater Accra, Volta, and Western, and high infant mortality regions (average deaths per 1,000 live births  > 61.3) were Central, Northern, Upper East, and Upper West. A series of post-estimation diagnostic tests reported in Table A1, Table A2, and Fig. A3 in the Appendix indicate no major diagnostic problems such as heteroskedasticity, non-normality, and multicollinearity.

**Table 2:** Empirical results. Dependent Variable: Infant mortality rate.

	(1) Full sample			(2) Low infant mortality regions			(3) High infant mortality regions		
	Coefficient	Std. Error	*P*-value	Coefficient	Std. Error	*P*-value	Coefficient	Std. Error	*P*-value
PHHE	−0.5415	0.2667	0.053*	−1.1841	0.4827	0.030**	−0.4615	0.4240	0.318
MBI	−1.4913	0.3602	0.000***	−1.2656	0.4515	0.016**	6.7689	1.6608	0.006***
PCLP	−0.8424	0.2079	0.000***	0.0101	0.4716	0.983	−2.7589	1.1534	0.053*
MYEW	−3.2712	6.1032	0.597	−12.990	5.6425	0.040**	25.6912	6.8517	0.009***
PPLQ	−0.5182	0.1147	0.000***	−0.4266	0.4855	0.397	−0.9626	0.4204	0.062*
PPHQ	0.1521	0.4426	0.734	−0.3908	0.6210	0.541	−12.9751	2.3968	0.002***
									
Adjusted R-squared	0.073			0.424			0.637		
Durbin-Watson stat.	3.1370			3.2007			2.1535		

*P*-value: ***, **, and * denotes respectively 1%, 5%, and 10% level of significance.

For the entire sample, an increase in access to electricity causes a statistically significant decrease in infant mortality rate. Specifically, a 10% increase in electricity access leads to a decline of 5.4 deaths per 1,000 live births. Also, longer birth intervals decreases the likelihood of dying before the fifth birthday. An increase in birth interval by one month reduces infant death by 1.4 per 1,000 live births. Children living with both parents also have a high probability of surviving. A 10% increase in the number of children living with both parents reduces infant mortality by 8 deaths per 1,000 live births. Women’s median years of education does not have a statistically significant impact on infant death. Households in the lowest wealth quintile experienced significant decline in infant mortality relative to households in the highest wealth quintile.

Because the sample average of 61.3 deaths per 1,000 live births is relatively high, we checked the robustness of the results by using the sub-Saharan African 2018 infant mortality rate of 58.3 deaths per 1,000 live births to split the sample into two (see Table A3 in the Appendix). Results did not differ when the sub-Saharan average was used to split the sample into two. The two exceptions are that electricity access causes a statistically significant decrease in infant death in both high and low mortality regions, and wealth quintiles had no statistically significant effect on infant death in both high and low mortality regions. As indicated in the methods section, the study could not also use the Millennium Development Goals (MDGs) target of 29 deaths per 1,000 live births or the Sustainable Development Goals (SDGs) target of 12 deaths per 1,000 live births [74]. This is because the minimum infant mortality rate in Ghana between 1993 and 2014 was 33 deaths per 1,000 live births. The SDGs and MDGs targets are far below the average infant mortality rate in Ghana. Hence, the use of the sub-Saharan benchmark of 58.3 deaths per 1,000 live births as robustness check.

When the data is split between low infant mortality and high infant mortality regions, the regions with low incidence of infant mortality experienced a decline in infant death with increased electricity access. Specifically, a 10% improvement in electricity access reduces infant mortality rate by 11.8 per 1,000 live births. In contrast, increased electricity access has no statistically significant impact on mortality in regions with high incidence of infant death. Birth interval significantly reduces infant death in low mortality regions but increases it in high mortality regions. The percentage of children living with both parents is inversely related with the likelihood of dying before the first birthday in both high and low infant mortality regions of Ghana. Specifically, high mortality regions saw a significant drop in infant death (27 deaths per 1,000 live births for a 10% increase in children living with both parents) compared to low mortality regions (0.1 deaths per 1,000 live births for a 10% increase in children living with both parents), although the decline in the latter region is not statistically significant. Women’s median years of education moves infant mortality rates in opposite directions for high and low regions. A 1-year increase in women’s education causes a statistically significant decline in infant mortality by 12.9 deaths per 1,000 live births in high mortality regions compared to a statistically significant increase of 25.6 deaths per 1,000 live births in low mortality regions. For the wealth quintiles, the estimates indicate that in regions with low incidence of infant mortality, wealth distribution has no statistically detectable impact on infant death. However, in regions with high incidence of infant mortality, both the wealthiest (population in the highest quintile) and the poorest (population in the lowest quintile) experienced significant decline in infant death, although the decline is much stronger for the wealthiest than it is for the poorest.

## 6 Discussions

The results reveal that significant electricity access gaps exist between the low and high infant mortality regions in Ghana and whereas the low infant mortality regions experience decline in infant death with increased electricity access, high mortality regions do not seem sensitive to increased electricity access. One plausible explanation is that because of chronic electricity access challenges in high infant mortality regions, residence have become depended on inefficient energy forms such as kerosene, biomass (wood, animal dung and crop waste), and charcoal. Consequently, when there are occasional increases in electricity access, these regions still rely on the inefficient energy forms which is known to have serious implications on maternal and infant health because women are usually responsible for cooking while caring for infants [43]. Besides, these regions are predominantly poor, and cost of electricity for many domestic uses may not be something many households can afford. Thus, the benefits of increased electricity access on infant health is dampen by the adverse effects from the use of inefficient energy forms.

Another plausible explanation for the absence of a statistically significant association between electricity access and infant death in high infant mortality regions is that mere increase in electricity access without requisite healthcare infrastructure will not necessarily improve health outcomes. As can be observed from Fig. S7 in the supplementary materials that shows the distribution of healthcare facilities across the 10 administrative regions of Ghana, most of the high infant mortality regions (shaded in red) have lower health facilities-to-population ratio relative to the low infant mortality regions (shaded in blue). Thus, policies that seek to promote better infant and maternal health outcomes through improved energy access should be implemented in tandem with healthcare infrastructure development policies.

Besides the inadequate infrastructure, the regions with high infant mortality do not have adequate healthcare professionals to staff the healthcare facilities. Fig. S8 in the supplementary materials shows the doctor-to-population ratio across the country. The high infant mortality regions are shaded in red and the low infant mortality regions in blue. Clearly the distribution disfavors the high infant mortality regions. On the extremes are Greater Accra region and the Upper West region. Whereas Greater Accra, the lowest infant mortality region, has 3,582 people per physician, Upper West, the region with the highest infant mortality rate, has 25,878 people per physician. These disparities signify that even if electricity access is improved, health outcomes will not necessarily improve without adequate healthcare infrastructure. Thus, improvement in the distribution of health professional, especially in high mortality regions, will improve service delivery to mitigate the incidence of infant mortality and other endemic health issues.

Regarding the control correlates, the finding that birth interval significantly reduces infant death in low mortality regions but increases it in high mortality regions supports the view that although the literature consistently finds short birth interval to be predictive of adverse infant outcomes, this is not universal. Some recent studies have found that, controlling for maternal factors, short birth interval does not elevate the risks of adverse neonatal and infant outcomes [75], [76], [77], [78]. In the case of Ghana, it could be that regions with high incidence of infant mortality do not practice carefully planned spacing or “good spacing” (such as family planning and proper provisioning for the pregnancy) which explains why longer birth interval does not reduce the likelihood of dying in those regions. Other studies have shown that longer birth interval per se is not a panacea to infant death but “good spacing” is the critical driver of low infant death [79], [80], [81]. Thus, sheer increases in birth interval without proper provisioning for the pregnancy will not reduce the likelihood of infant death. Furthermore, even if longer birth interval translates into better spacing, availability and access to critical healthcare infrastructure is crucial for birth interval to have the desired effect of reducing infant death.

That the percentage of children living with both parents reduces infant death is not surprising since households with both parents are more likely to initiate and follow through with pre-natal visits and other medical appointments, all of which improve the likelihood of infant survival [82]. In addition, families with both parents tend to have more resources to support healthy lifestyles, which in turn reduces adverse maternal and infant health outcomes [83], [84].

The women’s median years of education variable moves infant mortality rates in opposite directions for high and low regions. This could be driven by the observation that high mortality regions are rural and start with very low levels of education. Thus, a marginal increase in the years of education causes a strong decline in infant death. In contrast, low mortality regions are more urbanized and have high levels of education, but could over time suffer from *urban penalty* (higher mortality in urban areas) as urban economic and environmental conditions deteriorate in rapidly growing cities [85], [86]. The urban penalty effect typically operates through the labor force status of women. For instance, due to high unemployment rates, stress levels are higher among urban women, which attenuates the benefits of women’s education on infant mortality [87].

Since the median number of years of education completed by women do not capture the effects of different levels of education on infant death, the study breaks down education into four categories: no education, primary, secondary, and post-secondary education. Results show that in low mortality regions, infant death increases with no education but not statistically significant at the primary, secondary, and post-secondary education levels. In high mortality regions however, infant death decreases with primary and post-secondary education but not statistically significant with no education and secondary education (see Table A4 in the Appendix). These results imply that higher levels of women’s education bear more benefits in high mortality regions than in low mortality regions. This is consistent with the view that the more women are educated, the less the likelihood of adverse maternal and infant health outcomes [88], [89], [90], [91], [92], [93], although the effect is not as strong as some researchers and advocates have claimed [94].

For the wealth quintiles, the findings show that wealth distribution is inconsequential for infant death in low mortality regions, but in high mortality regions, both the wealthiest and the poorest experienced significant declines in infant death. However, the decline is much stronger for the wealthiest than it is for the poorest, and this could be attributed to the following reasons. The low mortality regions in Ghana are relatively urbanized and the health access gap between the wealthy and the poor is narrow compared to the rural/high mortality regions where the health access gap between the wealthy and the poor is wide. In urbanized/low mortality regions of Ghana, there are more government operated healthcare facilities that provide services to all irrespective of income level though the rich have slightly more access than the poor because of the former’s ability to afford additional sophisticated healthcare needs at private hospitals [95]. In contrast, only a handful of government healthcare facilities are located in rural/high mortality regions and these few existing ones are sparsely located. Thus, the rich (more so than the poor) in the rural communities are able to travel longer distances to where healthcare facilities are located. Consequently, in rural/high mortality regions, wealth differences exacerbate the health disparities between the rich and the poor. This finding supports the income inequality-health hypothesis and signifies that income inequality remains a core determinant of population health [96].

One limitation of the study is the focus on the previous 10 administrative regions in Ghana. An analysis using the new 16 administrative regions would have been yet another suitable approach to examine the relationship between electricity access and infant mortality, especially for most recent years. The data at hand, however, do not facilitate this type of estimation. The latest GDHS data is for 2014 whereas the new 16 regions took effect in 2019. Extending the analysis beyond 2019 to capture the new 16 administrative regions would require many more time series and cross-sectional data on the new regions than are available. Consequently, it is impossible to conduct an analysis using the new 16 administrative regions with the given data. As data on the new 16 regions in Ghana becomes available, the study can be replicated on these new regions. For other countries that have not introduced new subnational administrative regions, the method implemented in this study can also be replicated to continue to shed light on the disaggregated relationship between electricity access and infant mortality.

## 7 Conclusions and policy implications

This study investigated relationship between electricity access and infant mortality at the subnational level in Ghana, controlling for correlates such as birth interval, children living with both parents, women’s education, and income distribution. From the results and the analysis above, a number of targeted policy interventions can be implemented to mitigate the incidence of infant mortality in Ghana. The study recommends that the provision of reliable access to electricity is needed to improve infant mortality rates. However, policies that seek to improve access to reliable electricity should be implemented together with health infrastructure development policies, especially in the regions with high infant mortality rates. Absent of that, electricity access alone cannot have the desired effect.

In addition, policies, such as the national health insurance scheme (NHIS) and new-born care program (NCP), should be optimized to eradicate the service inequities between the rich and the poor. Furthermore, the wide disparities in electricity distribution and health service provision between the north and the south should be bridged. Relative to the south, the three regions in the north, i.e., Upper East, Upper West, and Northern, continue to have the highest incidence of infant mortality. They are also the regions with the least access to electricity and health services. This long-standing north–south divide reflected in the inequitable distribution of power supply, health facilities, clinical staff, and medical officers hampers the nation’s ability to adequately tackle the endemic infant mortality.

Finally, more health expenditure on technology is required because technology has the potential of transforming and enhancing health care provision in Ghana. The use of technology can speed up the adoption of cutting-edge medical procedures for infants and children. Other benefits of technology include disease outbreaks monitoring during pandemics, electronic patient records, appointment scheduling, video connect care, and remote surgery. However, the full benefits of technology will not be realized without robust cross-regional internet infrastructure and reliable access to electricity.

## CRediT authorship contribution statement

**Mikidadu Mohammed:** Conceptualization, Methodology, Software, Formal analysis, Supervision, Investigation, Data curation, Writing – original draft, Project administration. **Mathias Akuoko:** Conceptualization, Methodolody, Software, Formal analysis, Validation, Writing – review & editing.

## Declaration of Competing Interest

The authors declare that they have no known competing financial interests or personal relationships that could have appeared to influence the work reported in this paper.

## Figures and Tables

**Table A1:** Breusch-Pagan-Godfrey Heteroskedasticity Test Null hypothesis: homoscedasticity.

Full sample model			
F-statistic	0.4629	Prob. F(5,34)	0.801
Obs-R-squared	2.5495	Prob. Chi-Square(5)	0.769
Scaled explained SS	1.9827	Prob. Chi-Square(5)	0.851
Low infant mortality regions model			
F-statistic	0.3992	Prob. F(5,18)	0.842
Obs-R-squared	2.3961	Prob. Chi-Square(5)	0.792
Scaled explained SS	1.6680	Prob. Chi-Square(5)	0.892
High infant mortality regions model			
F-statistic	0.6571	Prob. F(5,10)	0.663
Obs-R-squared	3.9569	Prob. Chi-Square(5)	0.555
Scaled explained SS	2.0130	Prob. Chi-Square(5)	0.847

Prob: ***, **, and * denotes respectively 1%, 5%, and 10% level of significance.

**Table A2:** Jarque-Bera Normality Test. Null hypothesis: normal distribution.

	Test-statistic	*P*-value
Full sample model	1.9568	0.375
Low infant mortality regions model	1.6887	0.429
High infant mortality regions model	2.1545	0.341

*P*-value: ***, **, and * denotes respectively 1%, 5%, and 10% level of significance.

**Table A3:** Robustness check results using 2018 sub-Saharan Africa infant mortality rate as cut-off. Dependent Variable: Infant mortality rate.

	Low infant mortality regions			High infant mortality regions		
	Coefficient	Std. Error	*P*-value	Coefficient	Std. Error	*P*-value
PHHE	−2.7325	0.6730	0.003***	−0.8288	0.2336	0.006***
MBI	−3.3368	0.7822	0.002***	−1.8789	1.1989	0.151
PCLP	1.1991	0.7684	0.153	−2.4557	0.7434	0.009***
MYEW	−10.0567	5.5399	0.103	12.4583	6.1091	0.072*
PPLQ	−0.3402	0.2706	0.240	−0.4166	0.3054	0.206
PPHQ	0.0244	0.8389	0.977	0.2977	0.7825	0.712
						
Adjusted R-squared	0.476			0.176		
Durbin-Watson stat.	2.4773			2.6562		

*P*-value: ***, **, and * denotes respectively 1%, 5%, and 10% level of significance.

**Table A4:** Robustness check results using different levels of women’s education. Dependent Variable: Infant mortality rate

	Low infant mortality regions			High infant mortality regions		
	Coefficient	Std. Error	*P*-value	Coefficient	Std. Error	*P*-value
PHHE	−1.6312	0.7946	0.070	2.4607	0.8200	0.057
MBI	0.2569	2.1343	0.906	10.5279	6.1463	0.185
PCLP	−0.0455	0.4138	0.915	−1.4208	0.9603	0.235
NOEDU	0.3367	0.1128	0.015**	0.4468	1.7021	0.809
PRMEDU	0.2318	0.1582	0.177	−1.5403	0.4386	0.039**
SECEDU	−2.0403	1.6656	0.252	24.8441	10.6395	0.102
POSTSECEDU	4.4126	5.0511	0.405	−5.1783	1.7236	0.057*
PPLQ	−2.0305	0.9285	0.057	0.5284	0.1958	0.074*
PPHQ	0.4245	0.7151	0.567	−15.4367	8.7973	0.178
						
Adjusted R-squared	0.125			0.808		
Durbin-Watson stat.	3.2667			2.0123		

*P*-value: ***, **, and * denotes respectively 1%, 5%, and 10% level of significance.

## Appendix A

See Fig. A1, Fig. A2, Fig. A3, Table A1, Table A2, Table A3, Table A4
