# Supplementary material for: Subnational variations in electricity access and infant mortality: Evidence from Ghana
Source: Health Policy Open. 2021 Dec 2;3:100057. doi: 10.1016/j.hpopen.2021.100057 (PMC10297556; doi:10.1016/j.hpopen.2021.100057)
Supplement: Supplementary data 1 [file mmc1.docx]

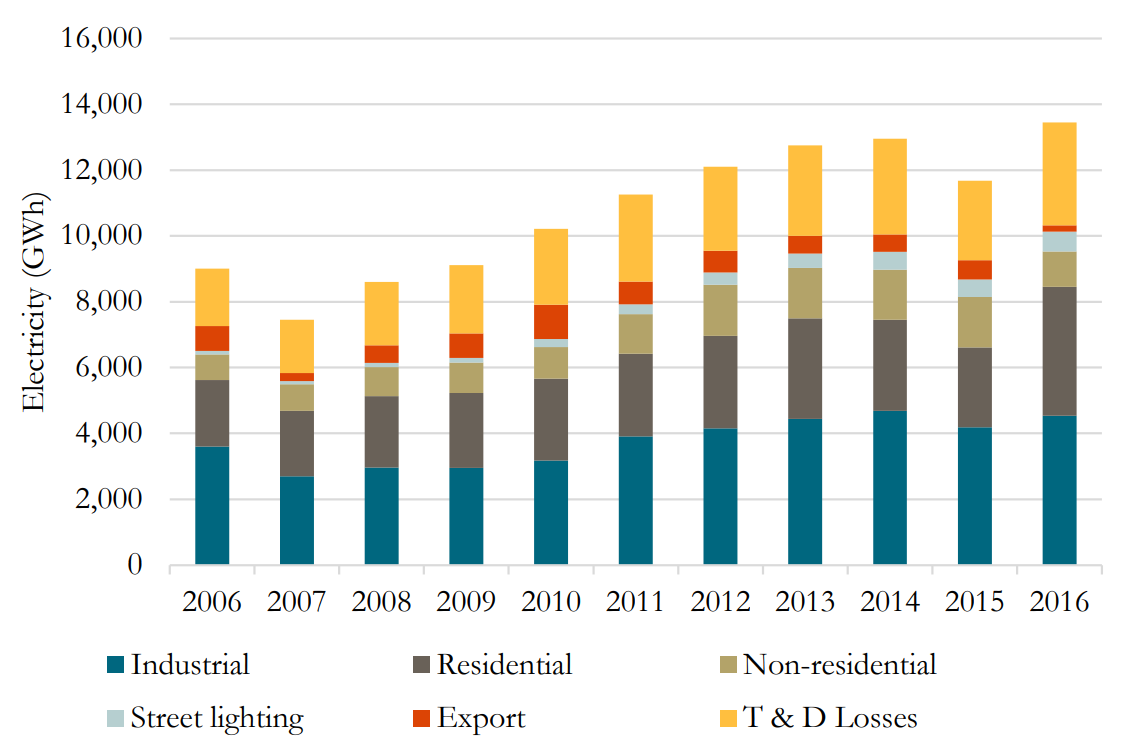


Figure S1: Ghana electricity consumption pattern

Source: Kumi (2017)

Ashanti Brong Ahafo Central Eastern Greater Accra

Northern Upper East Upper West Volta Western

Figure S2: Regional variation in electricity access and birth interval in Ghana (1993-2014).

Electricity access (blue line) is defined as percentage of households with electricity. Birth interval (red line) is defined as the median duration of the preceding birth interval (in months) for non-first births. Same data sources as in Figure 2.

Ashanti Brong Ahafo Central Eastern Greater Accra

Northern Upper East Upper West Volta Western

Figure S3: Regional variation in electricity access and children living with both parents in Ghana (1993-2014).

Electricity access (blue line) is defined as percentage of households with electricity. Children living with both parents (red line) is measured as percentage of de jure children living with both parents. Same data sources as in Figure 2.

Ashanti Brong Ahafo Central Eastern Greater Accra

Northern Upper East Upper West Volta Western


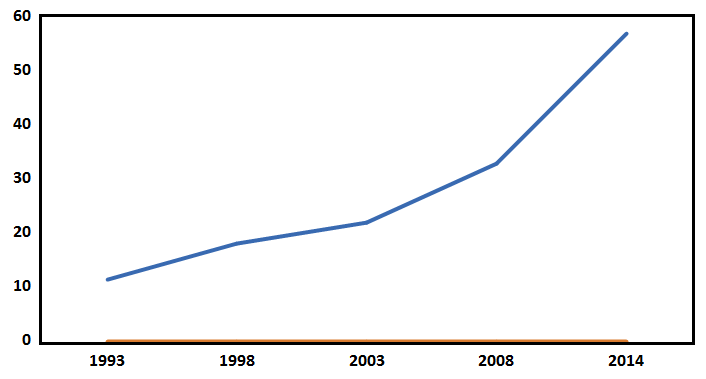

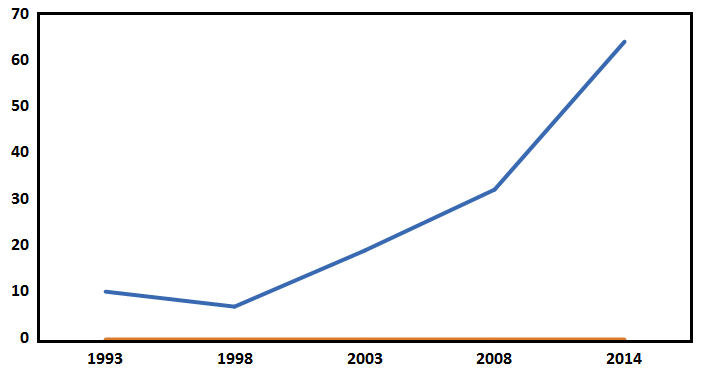


Figure S4: Regional variation in electricity access and women’s education in Ghana (1993-2014).

Electricity access (blue line) is defined as percentage of households with electricity. Women’s education (red line) is median number of years of education completed by women. Same data sources as in Figure 2.

Ashanti Brong Ahafo Central Eastern Greater Accra

Northern Upper East Upper West Volta Western

Figure S5: Regional variation in electricity access and population in lowest wealth quintile in Ghana (1993-2014).

Electricity access (blue line) is defined as percentage of households with electricity. Population in lowest wealth quintile (red line) is percentage of the de jure population in the lowest wealth quintile. Same data sources as in Figure 2.

Ashanti Brong Ahafo Central Eastern Greater Accra

Northern Upper East Upper West Volta Western

Figure S6: Regional variation in electricity access and population in highest wealth quintile in Ghana (1993-2014).

Electricity access (blue line) is defined as percentage of households with electricity. Population in lowest wealth quintile (red line) is percentage of the de jure population in the highest wealth quintile. Same data sources as in Figure 2.

Figure S7: Health facilities-to-population ratio by region (2016)

Blue = low infant mortality regions. Red = high infant mortality regions.

Source: Ghana Health Service

Figure S8: Doctor-to-population ratio by region (2016)

Blue = low infant mortality regions. Red = high infant mortality regions. Source: Ghana Health Service
